# Prise en charge d'une menace d'accouchement prématuré sur béance cervico-utérine au moyen d'un pessaire-cerclage obstétrical

**DOI:** 10.11604/pamj.2015.20.284.5847

**Published:** 2015-03-24

**Authors:** Erdogan Nohuz, Maël Albaut, Angélique Brunel, Nadine Champel, Julie Pellizzaro, Denis Gallot, Didier Lemery, Françoise Vendittelli

**Affiliations:** 1 Service de Gynécologie-Obstétrique, Centre Hospitalier de Thiers, Route du Fau, 63300 Thiers, France; 2 Service de Gynécologie-Obstétrique et Biologie de la Reproduction, CHU Estaing, 1, Place Lucie Aubrac, 63001 Clermont-Ferrand; 3 Service de Pédiatrie, Centre Hospitalier de Thiers, Route du Fau, 63300 Thiers, France; 4 Service de Pharmacie, Centre Hospitalier de Thiers, Route du Fau, 63300 Thiers, France

**Keywords:** Accouchement prématuré, menace d´accouchement prématuré, avortement tardif, cerclage cervical, pessaire obstétrical, incompétence cervicale, grossesse, premature delivery, preterm labor, late abortion, cervical cerclage, pessary obstetrical, cervical incompetence, pregnancy

## Abstract

Nous rapportons le recours efficace à un pessaire dans la prise en charge d'une menace d'accouchement prématuré. Une patiente de 28 ans, G2P1, ayant présenté une fausse-couche tardive à 20 semaines d'aménorrhée (SA) un an auparavant, bénéficiait d'un cerclage cervical à 15 SA. L’échographie endovaginale réalisée à 24 SA (sensation de pesanteur pelvienne) révélait un funnelling majeur et une longueur cervicale à 7 mm. Un pessaire obstétrical permettait la poursuite de la grossesse jusqu'au terme de 36 SA. Ce dispositif semble intéressant chez des patientes présentant une béance cervico-isthmique et chez qui un cerclage cervical s'est révélé inefficace.

## Introduction

Cause dominante de la morbi-mortalité périnatale, l'accouchement prématuré reste encore un problème de santé publique et sa prévention demeure un défi majeur pour les obstétriciens. L'incompétence cervicale apparait comme l'une des étiologies les plus fréquentes de la prématurité dont les facteurs de risque sont multiples: pertes fœtales survenant au deuxième trimestre, rupture des membranes avant 32 semaines d'aménorrhée (SA), traumatismes cervicaux, anomalies congénitales cervico-utérines [1–4]. Différentes alternatives, d'efficacité inconstante, sont proposées dans ce contexte: alitement, administration de progestérone par voie vaginale, cerclage du col et pessaire cervical [1, 5, 6]. Cet article relate le recours efficace à un pessaire obstétrical dans un contexte de récidive de menace d'accouchement prématuré sur une malformation utérine, survenue en dépit d'un cerclage chirurgical.

## Patient et observation

**Figure 1:**
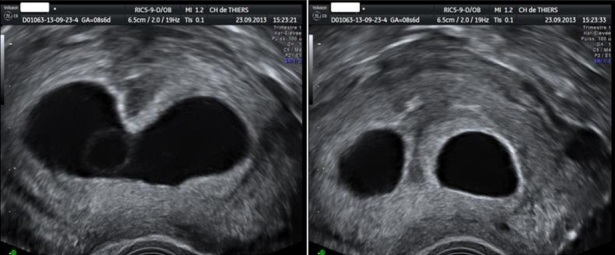
Malformation utérine à type de cloison partielle (fond arqué)

**Figure 2:**
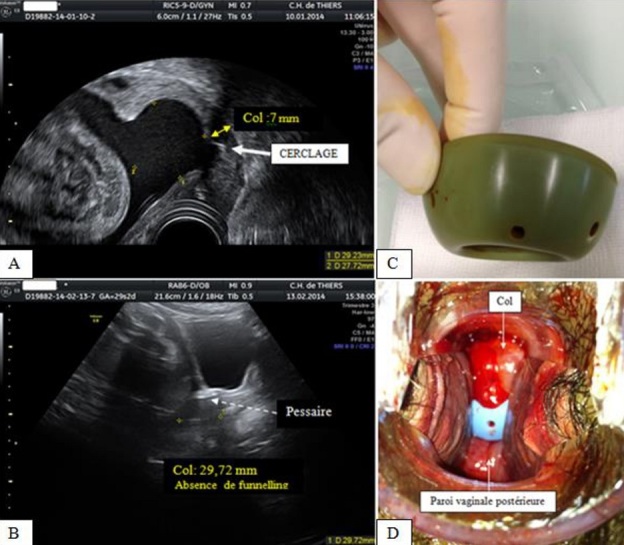
Aspects échographiques à l'admission et pessaire en place - vue du col pessaire in situ; échographie endovaginale à l'admission (24 SA): funnelling majeur(A); vue échographique sagittale médiane (voie sus-pubienne) à 28 SA: disparition du funnelling (B); pessaire obstétrical (C); aspect du col cerclé dans le pessaire obstétrical (examen au speculum à 36 SA) (D)

Une patiente de 28 ans, G2P1, ayant présenté un avortement tardif à 20 SA un an auparavant dans un contexte de malformation utérine à type de cloison partielle alliée à une béance cervico-isthmique, a bénéficié d'un cerclage cervical prophylactique (technique de Mac Donald-Hervet) à 15 SA (Figure 1). La malformation n'avait pas été prise en charge avant la grossesse en cours. L’échographie morphologique réalisée à 22 SA, sans signe d'appel particulier, a retrouvé une longueur cervicale à 28 mm. La patiente a consulté à 24 SA pour une sensation de pesanteur pelvienne. Le bilan a conclu en une menace d'accouchement prématuré avec, à l’échographie endovaginale, un funnelling majeur et une longueur cervicale efficace à 7 mm. Le fœtus présentait une bonne vitalité (poids estimé à 600g) et l'enregistrement cardiotocographique ne retrouvait pas de contraction utérine. La patiente a été hospitalisée, alitée en position de Trendelenburg (prévention des complications thrombo-emboliques par des bas de contention veineuse des membres inférieurs et un traitement par héparine de bas poids moléculaire à doses préventives). Le bilan biologique n'a retrouvé aucun stigmate d'infection pouvant évoquer une chorioamniotite. Un pessaire cervical obstétrical, perforé, en silicone, de type Arabin^®^ mesurant 65 mm de grand diamètre, 32 mm de plus petit diamètre et 25 mm de hauteur (Dr Arabin^®^ Cerclage Pessar, GmbH, Witten, Allemagne) a été alors mis en place, après obtention du consentement de la patiente (Figure 2). La surveillance échographique du col (réalisée par voie sus-pubienne) a objectivé une disparition du funnelling et une récupération d'une longueur cervicale supérieure à 25 mm. La grossesse s'est alors poursuivie sans problème majeur, autorisant une mobilisation progressive à partir de 28 SA puis la sortie. L'accouchement a eu lieu à 36 SA et demi, par césarienne compte-tenu d'une présentation du siège (version par manœuvre externe non tentée), permettant la naissance d'une fille pesant 3970g, sans malformation.

## Discussion

Le cerclage du col utérin apparaît actuellement comme la seule prise en charge efficace de l'incompétence cervicale. Réalisé à des fins prophylactiques, ce geste garde tout son intérêt dans cette indication, lorsque les antécédents obstétricaux le suggèrent et qu'une béance a été objectivée par un test à la bougie trois mois après un avortement tardif. Quant au cerclage thérapeutique, il est parfois indiqué lors de l'apparition de modifications du col à l’échographie (entre 16 et 22 SA). Cette surveillance s'adresse tout particulièrement aux patientes avec col court (conisation), malformation utérine et DES syndrome. Dans ces situations, la constatation d'une diminution de la longueur du col utérin (inférieure à 25 mm) ainsi que d'un élargissement de l'orifice interne (supérieur à 10 mm) est également évocatrice d'incompétence cervicale. La découverte, dans cette observation, d'une malformation utérine à type de cloison partielle alliée à une béance cervico-isthmique diagnostiquée au décours de l'avortement tardif ont plaidé pour la confection d'un cerclage cervical prophylactique. La iatrogénicité du cerclage cervical n'est cependant pas nulle (rupture prématurée des membranes, métrorragies, plaies vésicales, chorioamniotite, sepsis maternel) [6]. Le recours au pessaire cervical, qui représente une alternative séduisante, semble une voie encourageante. Un essai, publié récemment, a mis en exergue l'efficacité de ce dispositif (pessaire d'Arabin^®^). Dans cette étude prospective randomisée, les patientes enceintes (grossesses monofœtales) chez qui était réalisée une échographie systématique du deuxième trimestre entre 18 et 22 SA et qui présentaient une longueur cervicale inférieure à 25 mm étaient randomisées en deux groupes. L'un bénéficiait du pessaire tandis qu'aucun traitement prophylactique n’était entrepris pour les patientes du groupe témoin. Le groupe traité par le dispositif a présenté une réduction très significative de l'incidence des accouchements prématurés avant 34 SA comparativement au groupe témoin (Odds ratio: 0,18; IC à 95%: 0,08-0,37; p7]. La relative simplicité de mise en place et le coût modéré du pessaire soulèvent la question légitime de la mesure échographique systématique de la longueur du col utérin au deuxième trimestre de la grossesse afin de dépister les patientes asymptomatiques. Cependant, une autre étude vient contraster ces résultats. Pour les auteurs, le taux d'accouchement prématuré ne différait pas, que les patients aient bénéficié d'un pessaire ou pas [8]. Néanmoins, ce dernier essai ciblait moins les patientes à haut risque d'accouchement prématuré, ce qui peut représenter un biais d'inclusion. Ainsi, comme le soulignent Sentilhes et al., des études randomisées complémentaires doivent être menées avant de conclure en la nécessité d'une généralisation de cette évaluation échographique et la pose d'un dispositif en cas de modification cervicale [1].

Le mécanisme d'action du pessaire n'est pas totalement élucidé. Il reposerait sur la modification de l'angle cervico-utérin (rendant le col plus postérieur), la pression du canal cervical (protection du bouchon muqueux) ainsi que la diminution de la tension exercée par la présentation fœtale [1, 4, 5]. Le pessaire a pour objectif un soutènement et une bascule postérieure du col et non sa fermeture. Dans notre cas clinique, nous avons observé une disparition progressive du funnelling tandis qu'il nous était difficile de visualiser la bascule cervicale postérieure. Les indications du pessaire d'Arabin^®^, qui est doté d'une certification européenne, sont soit prophylactiques (grossesses gémellaires, pesanteur pelvienne, mode de vie astreignant), soit thérapeutiques (signes échographiques d'incompétence cervicale comme par exemple un col court ou ouvert au niveau de son orifice interne, entre 15 et 20 SA). Dans les instructions quant à l'emploi de ce dispositif, il est stipulé qu'un prélèvement vaginal à visée bactériologique est souhaitable préalablement à son placement, bien qu'il ne semble pas y avoir de modification de la flore bactérienne ni d'augmentation des chorioamniotites. A l'image du pessaire gynécologique, ce dispositif est élastique, ce qui facilite sa mise en place. Son insertion doit s'effectuer par un médecin, chez une patiente détendue, en position gynécologique, le bord courbe du dispositif (le petit diamètre) vers le haut afin que le diamètre le plus important puisse prendre appui sur le plancher pelvien. Le col utérin doit ainsi se situer à l'intérieur du petit diamètre, mais tout en s'assurant de l'absence de compression tissulaire excessive. Le recours à un lubrifiant peut faciliter le geste. La patiente doit alors se lever et effectuer quelques pas afin de vérifier sa bonne tolérance. La vérification ultérieure de la bonne position du pessaire sera réalisée par le toucher vaginal, l’échographie endovaginale devenant moins contributive. Notons que certains auteurs préconisent la voie périnéale ou la voie endovaginale modifiée (sonde dans le pessaire, au contact de la lèvre antérieure du col) [9]. Il convient de retirer le pessaire avant l'accouchement, ou vers 37 SA chez les patientes asymptomatiques. Ce retrait devra également s'effectuer en cas de rupture des membranes, de contractions utérines douloureuses ou de métrorragies importantes afin de limiter les risques d'infection et de dilacération cervicale. Rappelons qu'une augmentation des leucorrhées peut être observée, nécessitant rarement, semble-t-il, un retrait du dispositif pour lavage et remise en place [10].

## Conclusion

Le pessaire obstétrical pourrait représenter un traitement complémentaire voire une alternative thérapeutique ou préventive dans la prise en charge des menaces d'avortement tardif ou des menaces d'accouchement prématuré. Des études complémentaires restent un préalable nécessaire afin d'identifier sa place dans ces situations.

## References

[1] Sentilhes L, Descamps P, Legendre G (2014). Pessaire et prévention de l'accouchement prématuré. Gynecol Obstet Fertil..

[2] Iams JD, Romero R, Culhane JF, Goldenberg RL (2008). Primary, secondary, and tertiary interventions to reduce the morbidity and mortality of preterm birth. Lancet..

[3] Goldenberg RL, Culhane JF, Iams JD, Romero R (2008). Epidemiology and causes of preterm birth. Lancet..

[4] Liem S, Schuit E, Hegeman M, Biais J, de Boer K, Bloemenkamp K (2013). Cervical pessaries for prevention of preterm birth in women with a multiple pregnancy (Pro TWIN): a multicentre, open-label randomized controlled trial. Lancet..

[5] Liem SM, Van Pampus MG, Mol BW, Bekedam DJ (2013). Cervical pessaries for the prevention of preterm birth: a systematic review. Obstet Gynecol Int..

[6] Dubuisson J, Golfier F, Raudrant D (2009). Cerclage du col utérin: quelle technique, à quel terme, pour quelles patientes?. CNGOF, Mises à jour en gynécologie et obstétrique.

[7] Goya M, Pratcorona L, Merced C, Rodó C, Valle L, Romero A (2012). Cervical pessary in pregnant women with a short cervix (PECEP): an open-label randomized controlled trial. Lancet..

[8] Hui SY, Chor CM, Lau TK, Leung TY (2013). Cerclage pessary for preventing preterm birth in women with a singleton pregnancy and a short cervix at 20 to 24 weeks: a randomized controlled trial. Am J Perinatol..

[9] Goya M, Pratcorona L, Higueras T, Perez-Hoyos S, Carreras E, Cabero L (2011). Sonographic cervical length measurement in pregnant women with a cervical pessary. Ultrasound Obstet Gynecol..

[10] http://www.dr-arabin.de/.

